# Endothelial Cell Transition: Preliminary Data on Cross-Organ Shift from Brain to Liver

**DOI:** 10.3390/cells14191538

**Published:** 2025-10-01

**Authors:** Alexey Larionov, Luis Filgueira, Christian M. Hammer

**Affiliations:** Anatomy Unit, Section of Medicine, Faculty of Science and Medicine, University of Fribourg, Route Albert Gockel 1, 1700 Fribourg, Switzerland; luis.filgueira@unifr.ch (L.F.); christian.hammer@unifr.ch (C.M.H.)

**Keywords:** HGF, cell reprogramming, mouse brain endothelial cells (MBECs), mouse liver endothelial cells (MLECs)

## Abstract

**Background**: Endothelial cells (EC), crucial components of the vascular system, are adaptable cells that maintain homeostasis and respond to pathological events through structural and functional plasticity. Hepatocyte growth factor (HGF) is a multifunctional cytokine that has been demonstrated to have protective and disruptive influence on the blood barrier function. In endothelial biology, its role is also poorly characterized. The present study explores the impact of supraphysiological concentrations of HGF on mouse brain endothelial cells (MBECs), scrutinizing how it alters their integrity and morphology. **Methods**: Two groups of MBECs—control (CTR) and experimental (EXP)—were analyzed at two time points: early passage (p5) and late passage (p41). The EXP-groups (p5 and p41) were treated with HGF at a concentration of 4 µL/mL. Cellular morphology was assessed with brightfield microscopy; protein expression and localization of the tight junction marker (ZO-1) and the endothelial marker (Factor VII related antigen/von Willebrand factor, vWf) were analyzed using Western blotting, immunocytochemistry, and confocal microscopy. Intercellular barrier function was estimated via Transendothelial Electric Resistance (TEER) and Transendothelial Dextran Permeability (TEDP) assays. **Results**: *Microscopical analysis* demonstrated a change in the morphology of the MBECs from a longitudinal, spindle-like shape to a rounded, more spheroid, cobblestone-like morphology under high-dose HGF treatment. *Western blotting* revealed a progressive decrease of ZO-1 expression in the EXP-groups. The expression of vWf did not show significant differences. *Qualitative immunocytochemical staining*: vWf showed consistent expression across all groups. ZO-1 displayed a punctate, well-defined membrane and cytoplasmic localization pattern in the CTR-groups at p5 and p41. In contrast, the p5 EXP-group demonstrated a shift to a more diffuse cytoplasmic pattern. At p41, the EXP-group displayed a markedly reduced ZO-1 signal with no clear-cut membrane localization. *Confocal* analysis: ZO-1: punctate membrane-associated localization in CTR-groups at p5 and 41. The EXP-groups at p5 and p41 confirmed the diffuse cytoplasmic ZO-1 distribution. Phalloidin: well-organized actin cytoskeleton in CTR-groups, but rearrangement and stress fiber disorganization in the EXP-groups, especially at p41. The merged images confirmed reduced co-localization of ZO-1 with actin structures. *Barrier function*: TEER values dropped significantly in HGF-treated cells. TEDP to small and medium molecular weight dextran increased markedly under HGF treatment. **Conclusions:** Our data demonstrate that supraphysiological doses of HGF in an in vitro MBEC-barrier-like model disrupt TJ organization, leading to morphological changes and functional weakening of the MBEC-barrier-like structure, as shown by uncoupling between ZO-1/F-actin cytoskeleton, reduced TEER, and increased size-selective paracellular permeability (TEDP).

## 1. Introduction

Endothelial cells (ECs) are a highly dynamic and context-sensitive population exhibiting a remarkable degree of plasticity in response to local environmental cues. This sensitivity to the environment allows ECs to adapt their morphology and function in response to microenvironmental challenges, e.g., mechanical forces and tension [1,2,3], oxygen [4,5,6], cytokine exposure, and tissue-specific signaling molecules [7,8,9]. This responsiveness to external stimuli is observed across different vascular beds and physiological states and makes them promising targets for new strategies in tissue engineering and organ regeneration [10,11].

Moreover, they play a key role in the initiation and development of various pathological processes, where aberrant signals may initiate and drive endothelial dysfunction [7,8,12], inflammation [13,14], or maladaptive remodeling [15,16,17].

According to current understanding, the mechanisms of endothelial plasticity include two processes: endothelial transition and reprogramming. Endothelial transition constitutes a predominantly phenotypic shift through transient changes [18,19] Endothelial reprogramming, however, is a more stable conversion of ECs into distinctly different cellular subtypes across tissue or lineage boundaries [20].

There are two types of endothelial transition: endothelial-to-mesenchymal transition (EMT) and endothelial-to-hematopoietic transition (EHT) [21,22,23].

EMT is characterized by the ECs’ loss of specific endothelial markers (e.g., cluster of differentiation 31 [CD 31], tyrosine kinase with immunoglobulin-like and EGF-like domains [Tie2], von Willebrand factor [vWF], vascular endothelial cadherin [VE-cadherin]) [24,25], and the expression of mesenchymal markers (e.g., fibroblast-specific protein-1 [FSP1], alpha 2 smooth muscle actin [alpha-SMA], type I/III collagen, vimentin, and N-cadherin) [26,27]. This process coincides with profound changes in endothelial morphology, function, and specialization [28]. Furthermore, through EMT, the ECs may acquire new migratory and invasive properties, which facilitate tissue remodeling and alter the composition and structure of the local extracellular matrix [28,29].

EHT is primarily an embryological process in which ECs in the aorta–gonad mesonephros (AGM) region transiently acquire hematopoietic properties, giving rise to hematopoietic stem cells (HSCs), the primary precursors of all blood [30,31]. This tightly regulated transition is driven by key transcriptional factors, including runt-related transcription factor-1 (RUNX1), GATA binding protein (GATA2), and T-cell acute lymphocytic leukemia protein-1 (TAL1), which are responsible for the shift of ECs toward a hematopoietic fate [32,33,34,35]. Interestingly, the EHT transition may persist in some adult tissues; however, the role of this process in adults is questionable [36].

As opposed to trans-differentiation, endothelial reprogramming constitutes a process in which ECs undergo a phenotypic transformation through the alteration of their structural, molecular, and functional characteristics [37,38]. While trans-differentiation may be reversible, EC reprogramming involves a fundamental and irreversible change in cell identity from one specialized state to another, e.g., returning from a primitive to a pluripotent state, or a direct transition between distinct mature cell types [39,40]. Mechanistically, endothelial reprogramming is related to coordinated changes in gene expression [41,42], cytoskeletal organization [43,44], and junctional composition, often mediated by transcription factors [45,46,47], non-coding RNA [48] and epigenetic modulators [49,50].

Despite substantial progress in endothelial biology, the phenomenon of cross-organ endothelial reprogramming is poorly understood. Knowledge of the mechanisms that drive endothelial reprogramming is important not only for a deep comprehension of vascular adaptation, initiation, and development of wide-spectrum diseases, but also for identifying new therapeutic targets in tissue engineering, cancer, and regenerative medicine.

In the present study, we investigated whether mouse brain endothelial cells (MBECs) undergo phenotypic and functional changes in response to supraphysiological concentrations of human hepatocyte growth factor (HGF). Originally recognized as a powerful mitogen for hepatocytes, HGF also demonstrates pleiotropic effects in the nervous system and ECs [51]. HGF is abundantly present in liver tissue and is often elevated in the systemic blood circulation during liver disease [52,53]. It is a multifunctional cytokine that binds to and activates the C-met receptor and various downstream signaling pathways, e.g., the PI3K/Akt, MAPK, and STAT cascades [54,55,56,57,58,59]. As a result, it modulates the behavior of endothelial cells, including proliferation, migration, and survival [60]. Interestingly, in the context of endothelial barrier maintenance, HGF exhibits a functional duality, either promoting barrier stabilization [61] or, conversely, inducing cytoskeleton remodeling, following endothelial barrier breakdown and increasing barrier permeability [61].

The present study provides new insights into endothelial transition across organ boundaries in an MBEC-barrier-like model under supraphysiological concentrations of HGF.

## 2. Materials and Methods

### 2.1. Research Design

**Figure 1:**
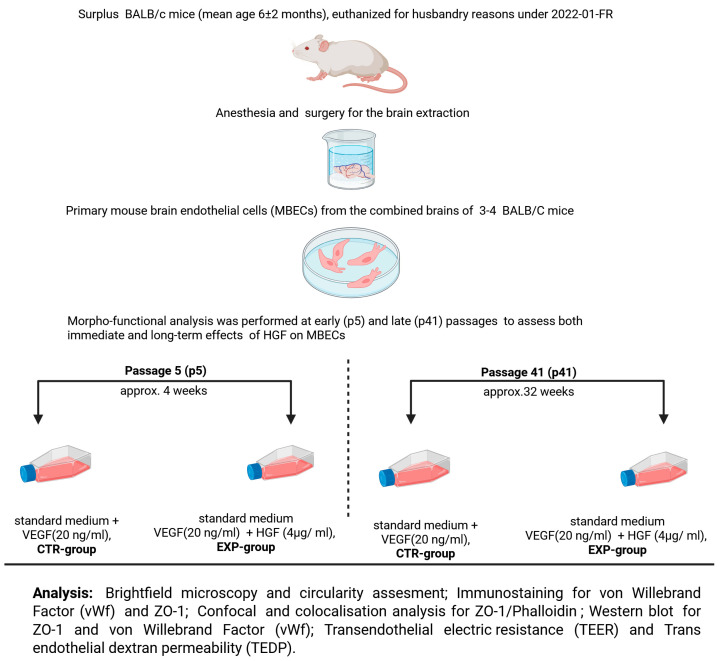
**Experimental setup for analysis of MBECs in control (CTR, no HGF) and experimental (EXP, with HGF) groups at different time points: passage 5 (p5) and passage 41 (p41)**.

Primary mouse brain endothelial cells (MBECs) were isolated from the brains of 3–4 BALB/c mice (mean age 6 ± 2 months). The MBECs were cultivated and expanded with standard medium (see below) containing vascular endothelial growth factor (VEGF, see Figure 1) until they reached passage 5 (p5). Then, high-dose hepatocyte growth factor (HGF) was added to the culture medium of the experimental group (EXP), while it was omitted in the control group (CTR). To investigate the time-dependent effect of high-dose HGF on MBEC morphology and barrier function, the cells were cultivated until they reached passage 41 (p41). The cells were tested for the expression of Anti-factor VIII/von Willebrand Factor (hereafter vWf) to verify their endothelial identity. The influence of HGF was investigated cross-sectionally (EXP vs. CTR) at p5 and p41, as well as longitudinally (p5 vs. p41) in the EXP and CTR groups. MBEC morphology was examined using brightfield and confocal fluorescent microscopy. To characterize the integrity and differentiation of tight junctions (TJs) within the monolayer, expression, distribution, and colocalization of Zonula occludens protein 1 (ZO-1) and fibrillar actin were investigated with immunofluorescence microscopy, confocal microscopy, and Western Blot. For a more direct assessment of the intercellular barrier function, trans-endothelial electric resistance (TEER) was measured, and the trans-endothelial dextran permeability (TEDT) assay was performed. Each experiment and every measurement were repeated at least 3 times.

### 2.2. Data Analysis

Each experiment/measurement was repeated at least 3 times. The exclusion criteria for mice were any genetic modifications or previously performed treatments affecting the cardiovascular system or other body systems via potential cross-reactivity. Data are shown as mean ± standard error of mean (SEM).

### 2.3. Reagents and Animals

Reagents used in the experiment were purchased from Sigma Aldrich (Merck, Darmstadt, Germany), Pan Biotech, Carpricorn Scientific, MACS Mitenyl Biotec. The BALB/C mice had a weight between 18 g and 28 g and were obtained from the local animal facility of the University of Fribourg, Switzerland. Organ extraction was conducted in accordance with husbandry license number 2022-01-FR, which covers the use of cells originating from surplus animals euthanized for husbandry reasons under the aforementioned license. This license was granted by the Cantonal Veterinary Office of Fribourg LSVW/SAAV; Euthanasia was performed by intraperitoneal injection of 150 mg/kg of Esconarkon (Sodium Pentobarbital) in 0.9% NaCl under conditions permitted by the FSVO (Federal Food Safety and Veterinary Office). The animals were kept under standard conditions and had free access to standard chow and tap water before euthanasia, followed by organ extraction.

### 2.4. MBEC Culture

Euthanized animals were perfused with a solution of 0.9% NaCl for 3–5 min. The primary culture of MBECs from mouse brains was prepared as previously described [62]. After the brain had been extracted and liberated from the meninges under sterile conditions, it was cut into small pieces, approximately 1 mm^3^ in size, followed by the extraction and purification of MBECs. To obtain a sufficient yield of MBECs for cell culture, the suspension from 3–4 mice brains was pooled.

MBEC cultures were grown for 4 days in Medium A, which was composed of DMEM/F12 (Sigma Aldrich M2279/Sigma Aldrich 51651C) with 10% of Fetal bovine supreme serum (FBS, PAN Biotech, P30-3031), HEPES sodium salt (≥99.5% titration, Roth, 6763.1) at 10 mM, Sodium bicarbonate at 13 mM (Sigma Aldrich 5761), mouse VEGF 20 ng/mL (MACS Mitenyl Biotec, 130-094-087), and 10% of commercial antibiotic/antimycotic mix (Carpricorn Scientific, cp23-6318). After 4 days, the medium was changed to “Medium B”, which contained all components of “Medium A” with only 5% of commercial antibiotic/antimycotic mix. The medium “C” (starvation medium), mediun which contained components of medium B without FBS, was used for Transendothelial electric resistance measurements (TEER). When the cultures reached 90% confluency (on days 5–7), the purified MBECs were split by brief treatment with a trypsin (0.05 *w*/*v*) and EDTA (0.02 *w*/*v*) solution. For MBECs cultivation, T25 and T75 flasks were used. All flasks were treated with rat tail collagen-I coating solution before cell seeding (Sigma Aldrich, 122-20). The splitting ratio was consistently 1:3.

The MBEC population was then divided into an experimental group (EXP) and a control group (CTR). The CTR-group contained only mouse VEGF at 20 ng/mL in the final volume (MACS Mitenyl Biotec, 130-094-087). The EXP group contained mouse VEGF at 20 ng/mL and human/mouse HGF at 4 μg/mL in the final volume (MACS Mitenyl Biotec, 130-103-437). The MBECs were treated with HGF starting from an early time point, passage 5 (p5, approximately 4 weeks of culture), until a late time point, passage 41 (p41, approximately 32 weeks), to induce the development of relevant morpho-functional alterations associated with the induced switching of MBECs to liver ECs. A comparison between the control (CTR, no HGF) and experimental (EXP, HGF-treated) groups was conducted after approximately one week of HGF incubation ensuing the respective cell splitting.

### 2.5. Brightfield Microscopy

Morphological changes were observed and photo-documented using brightfield microscopy. MEBCs were cultured as described in the cell culture section. Before the more detailed imaging of the cells, they were analyzed directly in the culture medium using a Nikon Eclipse TS100 inverted microscope equipped with a Canon DC7.4. V digital camera. Morphological characteristics analyzed include cellular morphology and multilayer formation. The morphology of the cells was monitored daily to observe morphological dynamics. Variations in confluency and cell density were observed at different magnifications. The focus was put on MBEC shape, cell boundaries, irregular clustering patterns, and intracellular details (nuclei and cytoplasmic granules).

As a control for the morphology assessment, we used the commercial Cadmec (dermal microvascular cells) cell line and primary mouse liver endothelial cells (MLECs). Cadmec cells were chosen as a comparative control for mouse brain endothelial cells, because they share the same spindle-shaped morphology and marker characteristics. Primary MLECs were chosen as positive control for the more spheroid morphology typical of hepatic endothelial cells.

All the images were taken under the same light and focus conditions for reproducibility. The images were archived with annotations for later qualitative and quantitative analysis. Per condition, 8–15 images were acquired; ≥100 cells/condition were segmented across multiple fields in each experiment.

### 2.6. Circularity Assessment

Cell morphology was quantitatively assessed using a circularity analysis of MBECs in brightfield images. Images were taken under standardized magnification and illumination conditions to ensure consistency across all experimental groups. Morphometric analysis was performed with ImageJ software (version 1.54p, National Institute of Health, USA). Images were converted to 8-bit grayscale, and then cells were segmented using the threshold tool. Individual cells were outlined manually using the polygon sectional tool, and the “Measure” function was used to obtain area and perimeter values for each cell, calculated by the formula:

$$Circularity=\frac{4\pi \times area}{{\left(Perimeter\right)}^{2}}$$

A minimum of 100 cells per experimental condition were measured across multiple fields of view. Circularity values range from 0 (completely irregular or elongated shapes) to 1 (perfect circle), serving as a quantitative indicator of morphological changes. Circularity values were averaged for each group, and statistical comparisons were performed to determine statistical differences. Normality of data was tested using the Shapiro–Wilk test. Circularity values for the p5 CTR- and EXP-groups were normally distributed, and comparison was made using an unpaired *t*-test, which showed no statistically significant differences. In contrast, the p41 CTR- and EXP-groups did not follow a normal distribution, and therefore, we applied the Mann–Whitney U test to assess the differences between conditions. Per condition, 8–15 images were acquired; ≥100 cells/condition were segmented across multiple fields in each experiment.

### 2.7. Endothelial Cell Immunostaining

Seeding of ECs: ECs were plated on the coverslips at a density between 7 × 10^4^–10 × 10^5^ cells per cover slip. The culture was grown according to standard culture protocol and with the solutions mentioned above at 37 °C with 5% CO_2_.

As a positive control for the qualitative assessment of MBECs in the CTR- and EXP-groups, we use the commercial Cadmec (Dermal microvascular cells) line. It was chosen due to its consistent expression of Markers such as vWf and ZO-1, providing a reliable reference for comparison with MBECs.

*Fixation of ECs:* After reaching the confluency of approximately 90%, the culture medium was removed, and samples were fixed by adding 500 µL of 3% paraformaldehyde (PFA) and subsequent incubation for 15 min at room temperature. Following fixation, the cells were washed thrice for 5 min with phosphate-buffered saline (PBS, Ph 7.4) to remove residual fixative.

*Permeabilization:* membrane permeabilization was achieved by adding 500 µL of 0.1% Triton X-100 to the coverslips, followed by 10 min of incubation at room temperature. The next step was to wash the confluent coverslips twice with PBS for 5 min to remove excess Triton X-100.

*Staining:* For identification of the target-specific endothelial cell markers, a primary Anti-factor VIII rabbit monoclonal antibody/von Willebrand Factor (vWf), Diagnostic Biosystems RP012, 1:1000 and a rabbit monoclonal anti-ZO-1 (Abcam ab276131, 1:1000) were diluted in PBS and incubated on the coverslips at room temperature for 2 h. As a secondary antibody donkey anti-mouse AF 488 (Jackson Immunoresearch, 715-546-151, 1:500 in PBS) was used for fluorescent labelling. Nuclear staining was performed with the Hoechst stain solution (Thermo Scientific H3570, 1:1000 in PBS) for 1 h in a dark chamber. After that, the wells were washed twice with PBS for 5 min to remove excess unbound secondary antibodies.

*Mounting:* At the final step, the coverslips were transferred to slides for microscopy, and the Vectashield mounting medium (Vector Laboratories, H-1000-10) was used to preserve the fluorescent signals.

### 2.8. Confocal Microscopy

Seeding and preparation of the cell culture were performed as described above. The vWF and ZO-1 immunostainings were compared between the CTR and EXP cell populations. Morphological changes and the organization of tight junctions (TJs) were analyzed with confocal microscopy. Cell–cell junctions were further assessed by conducting a colocalization analysis of anti-ZO-1 and phalloidin staining. For imaging of fibrillar actin, cells were stained with phalloidin to mark actin-rich areas, which are associated with TJs. The confocal images were obtained under constant laser intensity, pinhole size, and detector gain in all conditions to obtain reproducible results. ZO-1 was considered a marker for TJ integrity, and phalloidin staining provided the structural detail of the actin cytoskeleton. To evaluate the level of junctional alignment and structural continuity, the co-distribution of ZO-1 and F-actin was examined. 3D-reconstruction, thresholding, and segmentation tools in Imaris software (v.10.0.2) were used for quantitative colocalization analysis. For TJ integrity, Pearson and the correlation coefficient for object-based data were used. To ensure representative sampling, multiple fields of view were analyzed in every group. For each group, multiple fields of view (more than 10) were analyzed per experiment under typical acquisition settings. Quantifications of colocalization data were shown as mean ± standard error of mean (SEM).

### 2.9. Protein Quantification

Protein extraction was performed using 100 µL of RIPA lysis buffer supplemented with protease and phosphatase inhibitors to ensure protein integrity. Confluent MBECs were washed twice with cold PBS and lysed directly on the plate by adding ice-cold lysis buffer. The lysate was incubated on ice for 30 min and periodically agitated to ensure complete cell disruption. Following lysis, samples were centrifuged at 300 rcf for 5 min at 4 °C to pellet the cell debris. The supernatant containing soluble protein was collected, and protein concentration was determined using the BSA assay.

All protein concentrations from cellular homogenates were determined using a colorimetric protein assay based on the biuret reaction (Pierce, BCA Protein Assay Kit, Thermo Scientific, Switzerland). The color complex specific to this method intensifies proportionally with the concentration of the reacting protein, affecting its color intensity. To carry out the biuret reaction, all samples were incubated at 37 °C for 30 min and then cooled to room temperature. The albumin standard was used to establish a linear calibration curve, and the corresponding protein concentration was calculated by spectrophotometrically measuring the extinction of the samples at 562 nm with Thermo Scientific, Multiscan Sky High.

### 2.10. Western Blot Analysis

The separation of the proteins within the different cellular homogenates was performed by the SDS–Page method. For this purpose, the samples were dissolved in Laemmli buffer and completely denatured for 5 min at 95 °C. The molecular weight was determined with a pre-stained protein ladder (Thermo Fisher Scientific Inc., USA).

Membranes were blocked with 5% skim milk in Tris-buffered saline containing 0.1% Tween-20. The following antibodies were used: Anti-ZO-1 (1:1000, Abcam, ab276131, rabbit); Anti-factor VIII rabbit monoclonal antibody/von Willebrand Factor (vWf, Diagnostic Biosystems, rabbit, RP012, 1:1000); Tubulin (1:2000, Protein Tech, cat. Nr 66031, mouse) in 1% skimmed milk. Incubation was performed overnight at 4 °C.

The next day, the membranes were washed with 0.5% Tween/TBS solution and incubated with peroxidase-conjugated anti-mouse (R&D Systems, HAF 007) and anti-rabbit (HAF 008) immunoglobulin secondary antibodies for 2 h, followed by three rounds of blot washing. Immunoreactive bands were detected using an enhanced chemiluminescence kit (Amersham ECL Select Western Blotting Detection Reagent, GE Healthcare, London, London, UK) followed by subsequent application of Syngene G: Box Chemi XX6, GeneSys and GeneTools software (Cambridge, UK).

Tubulin (52 kDa) was used as a loading control to compensate for variations in protein loading, and band intensities were normalized to tubulin for accurate quantification in Western blot analysis. Densitometric quantification of protein band intensity was performed using ImageJ software, all data were normalized to Tubulin abundance, and control values were set as 100%.

### 2.11. Transendothelial Electric Resistance (TEER)

For this assay, Brand 24-well cell culture inserts with PET membranes (pore size 0.4 µm, Sigma Aldrich, Br782710) were used. The inserts were pre-treated with rat tail collagen-I solution (Sigma Aldrich, 122-20). MBECs (2.5 × 10^5^ cells/cm^2^) were seeded on the PET membranes without astrocytes/pericytes co-culture, cAMP/hydrocortisone induction, or applied shear stress conditions known to raise TEER in other BBB (Blood–Brain Barrier) platforms. The TEER measurements were conducted using the Millicell^®^ ERS-2 Electrical Resistance System.

In the first step of endothelial monolayer cultivation, Medium B was applied and changed every 2 days. The assessment of TJ integrity (endothelial monolayer formation) was performed every 2 days and stopped when the independence values of the CTR-group reached the target values of 100 Ω°Cm^2^ (approximately at 4–7 days after cell seeding). In the second step, “Medium B” was replaced by “Medium C”, for overnight incubation. In the third step, HGF was added to the experimental monolayer. The TEER measurements were conducted every day to estimate the monolayer evolution in the presence of HGF in the medium (7–14 days). Empty Brand^®^ collagen-treated inserts (without cells) were used to measure background resistance. These values were subtracted from the resistance values of the two experimental groups. Results are reported in Ω°Cm^2^.

Electrophysiological transepithelial voltage is a measure of TJ integrity and was calculated using the equation:

$$TJ\;integrity=\frac{\frac{Vte(mV)}{TEER(ohm)}\times 1000}{0.3}$$

(transepithelial electrical resistance (TEER), transepithelial electrical voltage (Vte), 0.3 cm^2^-area).

Values are reported as mean ± standard error of mean (SEM), from ≥3 independent experiments, and blank-insert resistance was subtracted.

### 2.12. Transendothelial Dextran Permeability (TEDP)

To estimate the macromolecular flux through the endothelial monolayer and assess TJ functionality, we used fluorescein isothiocyanate (FITC)-labeled Dextran of different molecular weights—70,000 (Sigma Aldrich, FD70 S), 10,000 (Sigma Aldrich, FD10S) and 4000 (Sigma Aldrich, FD4)—at a final concentration of 0.5 mg/mL.

The method was carried out as described previously [63,64]. After cell monolayer formation, FITC-labeled Dextran was added to the apical compartment of the insert filters and incubated for 2 h at 37 °C, protected from light. Following the incubation period, medium from both the apical and basolateral compartments was collected and the fluorescence intensity of samples was measured using black flat-bottom microplates (Corning^®^, 96 well CLS3615). The permeability coefficient Papp (cm/min) was calculated with an excitation wave of 490 nm and an emission of 520 nm according to the formula below:

$$Papp=\frac{△Q}{△t}\times \frac{1}{A\times C0}$$

where ΔQ/Δt is the rate of permeabilization of dextran µg/min across the endothelial layer, A is the surface of the cell layer (Cm^2^), and C0 is the initial dextran concentration µg/mL, which was applied to the apical part of the cell layer.

Values are mean ± standard error of mean (SEM) from 3 independent experiments; Papp calculated as specified.

### 2.13. Statistical Analysis

The statistical analysis was performed with the GraphPad Prism 10.4.1 software. Data distribution was first assessed with the Shapiro–Wilk normality test. For group comparisons, non-parametric tests were applied as appropriate: the Mann–Whitney U test for two group comparisons, and the Kruskal–Wallis test for multiple groups. Categorical variables were analyzed using the chi-squared test. A *p* value of <0.05 was considered statistically significant. The results are presented as the mean ± standard error of mean (SEM) of at least three replicates of each experiment.

## 3. Results

### 3.1. MBEC Morphology and Cytoarchitecture

#### 3.1.1. Western Blot

**Figure 2:**
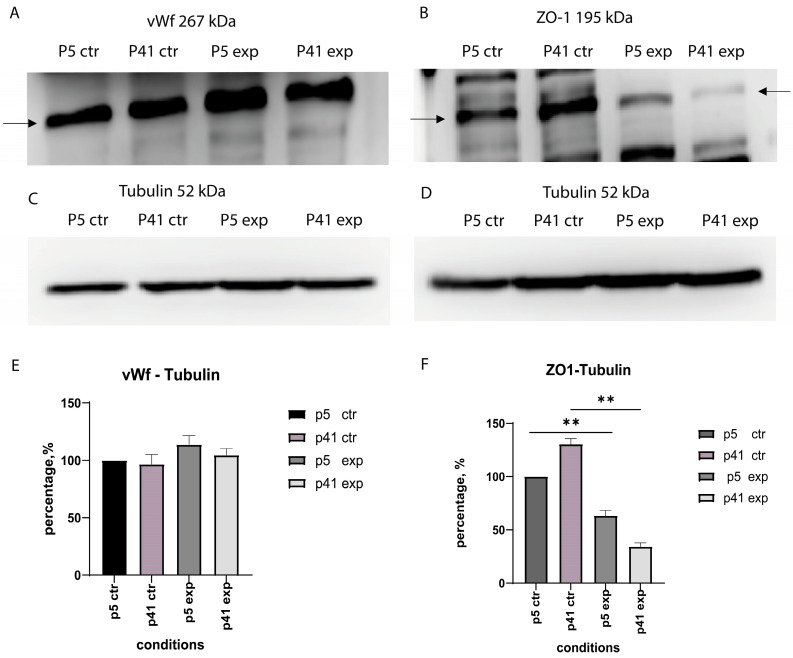
**Endothelial marker expression in MBECs under HGF treatment and at different passages.** (**A**) Western blot detection of von Willebrand factor (vwF), 267 kDa in control (CTR) and experimental (HGF) groups at early (p5) and late passage (p41). (**B**) Western blot detection of tight junction protein ZO-1, 195 kDa in control (CTR) and experimental (HGF)-groups at early (p5) and late passage (p41); (**C**,**D**) Western blot detection of Tubulin (52 kDa) for vWf and ZO-1: in control (CTR) and experimental (HGF)-groups at early (p5) and late passage (p41); (**E**,**F**) Quantification of Western blot band intensities normalized to Tubulin: (**E**) Densitometric analysis of vWf/Tubulin expression; (**F**) Densitometric analysis of ZO-1//Tubulin expression. Data are Data shown as mean ± standard error of mean (SEM), *n* number of independent experiments ≥ 3. Statistical analysis performed using a Mann–Whitney U test, ** *p* < 0.05.

As can be seen in Figure 2A, vWf (267 kDa) was expressed in the CTR group in both p5 and p41, which indicates an endothelial phenotype. Notably, the data suggests a minor trend toward increased expression of vWf after HGF treatment EXP groups of p5 and p41 (Figure 2A). However, this difference did not achieve statistical significance.

HGF treatment resulted in a decrease of ZO-1 expression in the EXP-groups (HGF). In p5 cells, the HGF-induced reduction in ZO-1 expression was moderate, whereas in p41 cells, it was very pronounced, with ZO-1 being almost undetectable after HGF treatment (Figure 2B).

#### 3.1.2. Brightfield Microscopy

**Figure 3:**
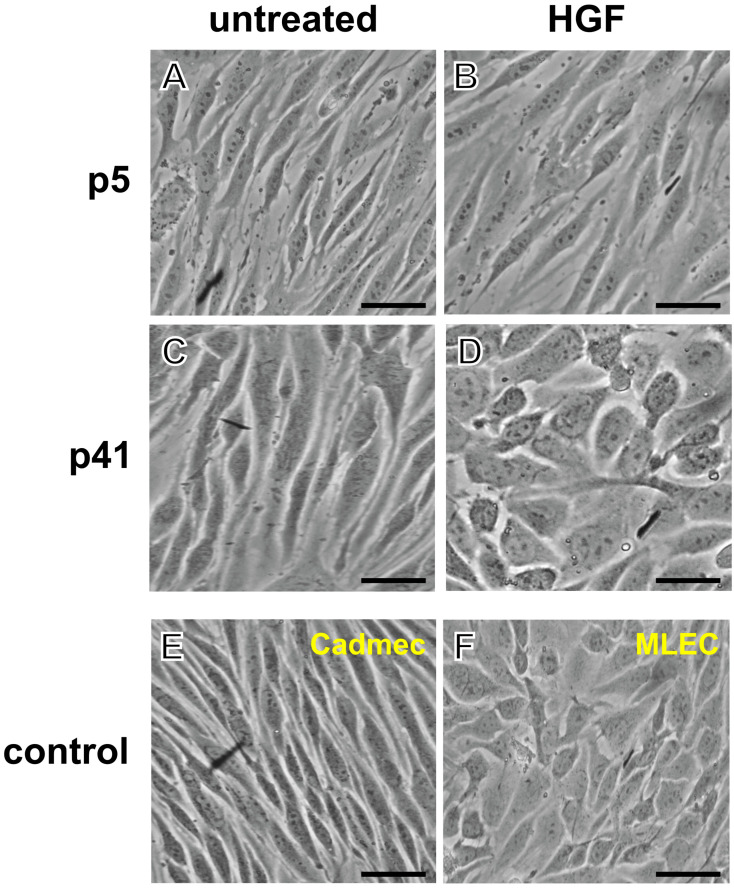
**Morphological changes of MBECs induced by HGF treatment.** (**A**–**F**) Brightfield microscopy images under different conditions: (**A**) MBECs, untreated control-group (CTR-group, No HGF), early, passage 5 (p5); cells display the typical spindle-shaped morphology. (**B**) MBECs, experimental (EXP-group, with HGF), early passage 5 (p5); cells display the typical spindle-shaped morphology. (**C**) MBECs, Control-group (CTR-group, No HGF), late, passage 41 (p41); cells display the typical spindle-shaped morphology (**D**) MBECs, experimental (EXP-group, with HGF), late passage 41 (p41); cells exhibit a spheroid morphology typical of liver endothelial cells. (**E**) Cadmec (Dermal microvascular cells): morphological control for CTR group (spindle-shaped); (**F**) Mouse liver endothelial cells: morphological control for EXP-group (spheroid shape); Sample size of independent experiments *n* ≥ 3. Scale Bar is 50 µm.

**Figure 4:**
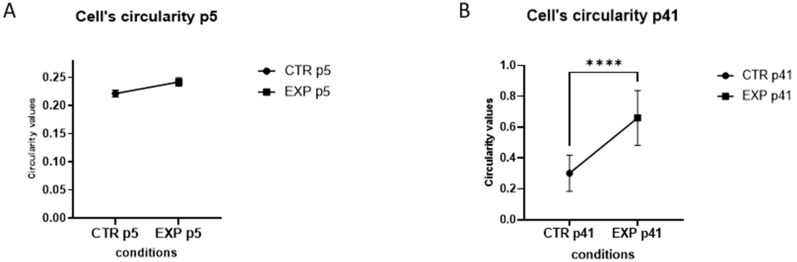
**Quantitative Analysis of circularity under HGF treatment.** (**A**) Quantification of cellular circularity in CTR and EXP groups of MBECs at passage 5 (p5). No statistically significant difference in circularity. (**B**) Quantification of cellular circularity in CTR and EXP groups of MBECs at passage 41 (p41). Difference in circularity shows a high statistical significance. Data shown as mean ± standard error of mean (SEM), ≥100 cells/condition analyzed. Statistical analysis: Mann–Whitney U test, **** *p* < 0.0001.

In the CTR-group, p5 MBECs presented a typical, elongated shape with tight monolayer alignment (Figure 3A). Compared to that, the EXP-group p5 of MBECs appeared slightly less elongated (Figure 3B). However, quantitative circularity analysis showed no statistically significant differences between EXP- and CTR-groups (Figure 4A). CTR-group p41 of MBECs demonstrated normal spindle-shaped morphological characteristics despite the high passage number (Figure 3C). In stark contrast, the late passage MBECs (p41) displayed a pronounced morphological transformation under HGF treatment. First of all, they obtained rounded, compact cellular shapes. Second, they displayed a disrupted monolayer organization when compared to the CTR-group of p41 (Figure 3D). This phenotype alteration was confirmed quantitatively by circularity analysis, revealing a statistically significant increase in circularity values in HGF-treated p41 cells compared to passage-matched controls (Figure 4B). Notably, p41 cells treated with HGF (Figure 3D) underwent complete phenotypic transformation, transitioning from their original brain endothelial cell morphology to a morphology more typical of liver endothelial cells (LECs). This morphological alteration was statistically validated by a robust increase in circularity values of the EXP group vs. the CTR group (Figure 4A,B, **** *p* < 0.0001). The morphology of primary culture MBECs was compared with that of the commercial Cadmec (Dermal microvascular endothelial cells) cell line used as a control (Figure 3E). The morphology of the EXP-group was compared to MLECs for assessment of the phenotypic changes that occurred under the HGF treatment (Figure 3F).

#### 3.1.3. Endothelial Cell Immunostaining

The expression and localization of vWf and ZO-1 proteins in MBECs were assessed by immunofluorescent staining.

**Figure 5:**
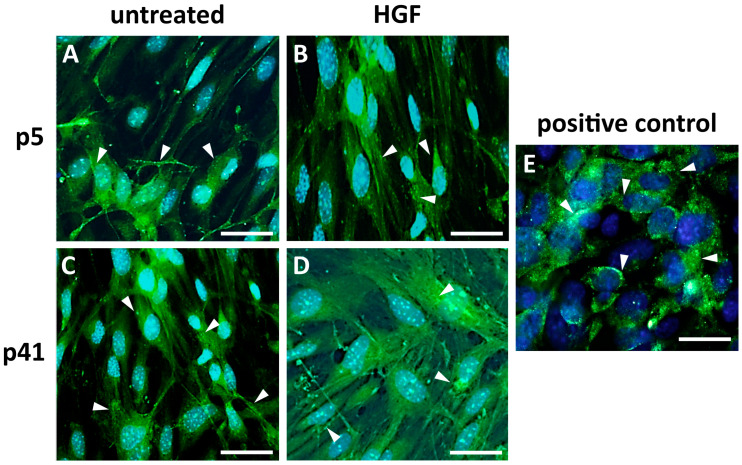
**Immunocytochemical analysis of vWf (green) expression in MBECs with and without HGF treatment.** (**A**–**C**) Normal and pronounced staining of Weibel-Palade bodies in untreated p5 and p41 MBECs as well as in HGF-treated MBECs at p5. (**D**) Increased cytoplasmic intensity of vWf staining partially independent of Weibel-Palade bodies. (**E**) Cadmec cells were used as a positive control. Blue: Hoechst nuclear staining; White arrows: Weibel-Palade bodies; Sample size of independent experiments *n* ≥ 3. Scale bar: 50 µm.

*vWf expression* (Figure 5): Cadmec cells were used as a positive control (Figure 5E). The untreated CTR-groups of both early-passage p5 (Figure 5A) and late-passage p41 MBECs (Figure 5C) demonstrated the punctate cytoplasmic vWf staining, consistent with Weibel-Palade bodies (indicated by white arrows). Following HGF treatment (EXP-groups, Figure 5B,D), both early passage (p5) and late-passage (p41) of MBECs remained positive for vWf. Notably, late passage MBECs (p41) in the HGF-treated group displayed enhanced signal intensity with expanded cytoplasmic distribution (Figure 5D).

**Figure 6:**
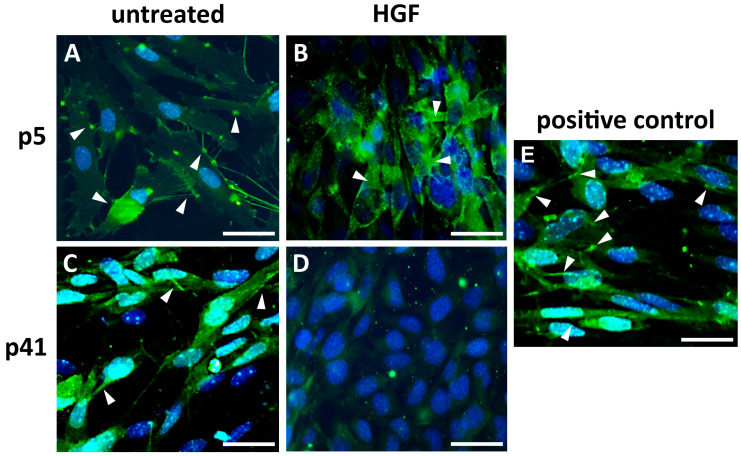
**Immunocytochemical analysis of ZO-1 expression (green) in MBECs.** (**A**) Focal detection of ZO-1 at the cell borders of untreated p5 MBECs. (**B**) Disrupted pattern of ZO-1 immunoreactivity after HGF treatment in p5. The immunosignal has partially shifted into the cytoplasm and appears incongruent with the location of tight junctions. (**C**) Weakened and partially non-focal detection of ZO-1 in untreated p41 MBECs. (**D**) HGF-treated p41 MBECs have almost lost their ZO-1 immunoreactivity. (**E**) Cadmec cells as positive controls. White arrows: ZO-1 junctional expression Blue: Hoechst nuclear staining; Sample size of independent experiments *n* ≥ 3. Scale bar: 50 µm.

*ZO-1 expression* (Figure 6): Cadmec cells were used as a positive control (Figure 6E). In the untreated CTR-group, the early-passage (p5) ZO-1 immunolocalization was detected as continuous, well-defined dots at the cell borders (Figure 6A). The early passage (p5) of the EXP group had a weaker and more fragmented ZO-1 distribution pattern with partial disruption from the intercellular junction complex (Figure 6B). ZO-1 expression in late-passage MBECs (p41) of the CTR-group was still well defined but reduced and less continuous (Figure 6C). The ZO-1 signal is almost absent, and the cell-cell junctional pattern is almost lost in the EXP-group of late-passage (p41) MBECs (Figure 6D).

#### 3.1.4. Confocal Microscopy

**Figure 7:**
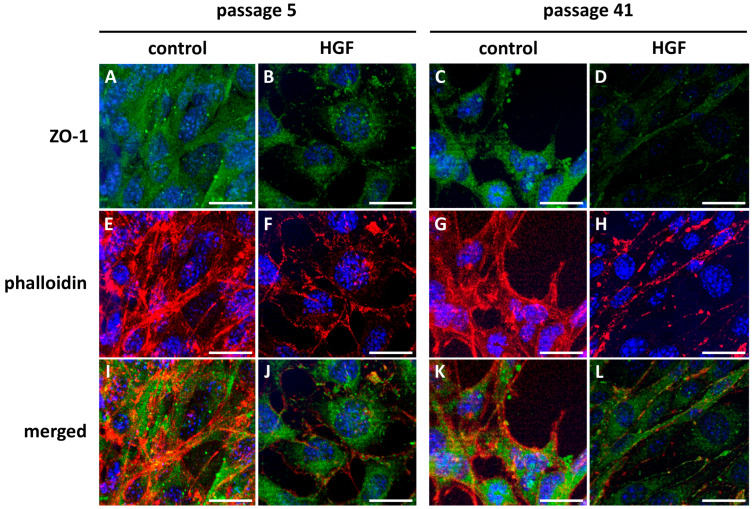
**Confocal Immunofluorescence analysis of ZO-1 and phalloidin colocalization in MBECs.** P5 untreated (**A**,**E**,**I**): marked colocalization of ZO-1 and actin at the membranes, indicating intact tight junctions. P5 HGF-treated (**B**,**F**,**J**): partial disruption of ZO-1/actin colocalization. P41 untreated (**C**,**G**,**K**): less pronounced ZO-1/actin colocalization as compared to p5 untreated. P41 HGF-treated (**D**,**H**,**L**): substantial loss of ZO-1/actin colocalization after prolonged HGF treatment; Sample size of independent experiments *n* ≥ 3. Scale bar: 50 µm.

Confocal microscopy colocalization analysis revealed a junctional disruption caused by HGF-treatment. In the untreated p5 CTR group (Figure 7A,E,I), ZO-1 localized at intercellular borders in a continuous manner, strongly colocalizing with well-organized actin fibers at the cortical actin band. This observation indicated that TJs were intact. Numerous yellow puncta in the merge and colocalization panels indicate that ZO-1 and F-actin were colocalized at these junctions, providing further evidence of close spatial association (Figure 7I). The cell morphology is in concordance with the classic spindle-like shape.

**Figure 8:**
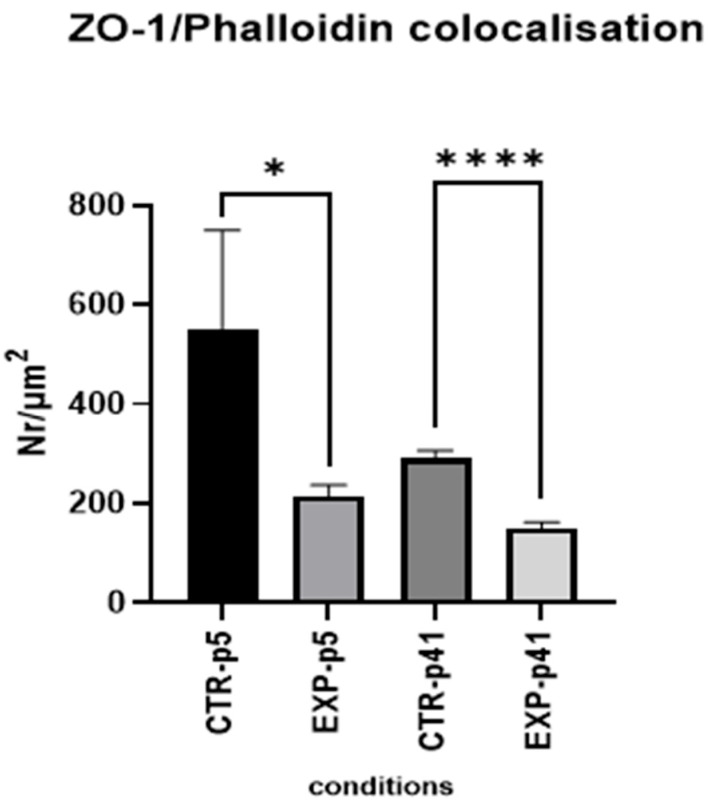
**Colocalization analysis of ZO-1 and phalloidin.** Quantification of ZO-1 and phalloidin colocalization, expressed as the number of colocalization events per µm^2^. Data are presented as mean ± SEM. Statistically significant decrease in colocalization after HGF treatment in MBECs; Sample size of independent experiments *n* ≥ 3 with multiple fields/group (≥10). Statistical analysis was performed using the Mann–Whitney U test * *p* < 0.05, **** *p* < 0.0001.

The HGF-treated p5 MBECs (Figure 7B,F,J) demonstrated a slightly disrupted junctional pattern, as ZO-1 appeared more cytoplasmic and less organized at the membrane (Figure 7B). The F-actin displayed a more stress fiber-like and disorganized configuration (Figure 7F), with significantly decreased colocalization of ZO-1 and phalloidin staining (Figure 7J). The quantitative analysis of colocalization density (Figure 8) showed a significantly reduced overlap between ZO-1 and phalloidin in EXP-group p5 cells treated with HGF compared to controls (* *p* < 0.05), suggesting early signs of junctional disassembly and initial cytoskeleton reorganization.

In the CTR-group p41, the colocalization of ZO-1/phalloidin was lower as compared to the CTR-group p5 (Figure 7K). ZO-1 was present, but distributed in a more diffuse way (Figure 7C), while actin displayed a less uniform expression pattern (Figure 7G).

After HGF treatment, we observed more dramatic structural alterations in EXP-group p41 (Figure 7D,H,L). During the long treatment, ZO-1 lost its association with cell junctions (Figure 7D), and actin staining demonstrated a shift from cortical to contractile cytoskeleton (Figure 7H). The colocalization signal was minimal, as shown in the colocalization merge image (Figure 7L). Quantification confirmed a statistically significant decrease in colocalization compared to the CTR-group (*p* = 0.41, Figure 8).

### 3.2. MBEC Physiology and Barrier Function

#### 3.2.1. TEER Measurements of MBECs Monolayers

**Figure 9:**
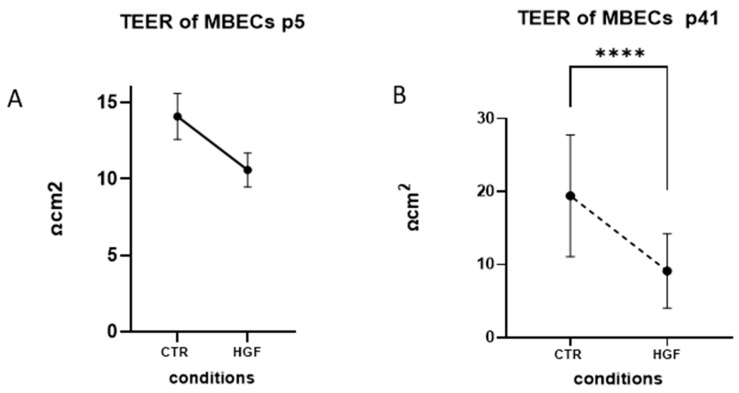
**Transendothelial electrical resistance (TEER) in MBEC monolayers cultured with or without HGF.** (**A**) TEER measurements in MBECs at early passage (p5) and (**B**) late passage (p41) in CTR-and EXP-groups. Data are presented as mean ± standard error of mean (SEM), sample size of independent experiments *n* ≥ 3. Statistical significance was determined using unpaired *t* tests; **** *p* < 0.0001.

As seen in the TEER data (Figure 9A), the CTR-group p5 had an apparently intact monolayer with stable TEER values of 14–15 Ω*cm^2^, representing intact TJs. After the HGF treatment, we observed a marked, but statistically insignificant, decrease in the TEER reading (approximately 11 Ω*cm^2^). This fact is an indicative initial step toward barrier destabilization of MBECs under the HGF treatment. In EXP-group p41, the effect of HGF was more dramatic, as TEER values dropped significantly after the treatment in a time-dependent manner, from around 20 Ω*cm^2^ in the CTR-group to under 10 Ω*cm^2^ in the EXP-group (Figure 9B, **** *p* < 0.0001). Such a massive reduction of TEER demonstrated a drastic HGF-induced barrier integrity dysfunction in the late-passage MBECs population.

#### 3.2.2. Size-Selective Transendothelial Dextran Permeability (TEDP) in MBECs Monolayers

**Figure 10:**
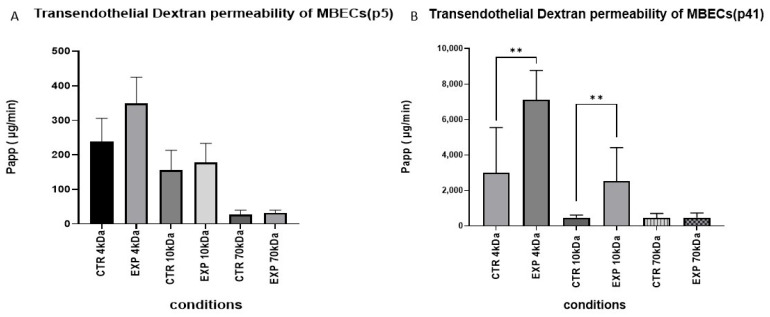
**Transendothelial dextran permeability (TEDP) in MBECs monolayers at p5 and p41 in CTR- or EXP-groups using fluorescently labeled dextran molecules of different molecular weights (4, 10, 70 kDa):** (**A**) TEDP (µg/min) at MBECs p5 in CTR and EXP-groups; (**B**) TEDP (µg/min) at MBECs p41 p5 in CTR and EXP-groups. Statistically significant increase in trans-endothelial permeability of 4 kDa and 10 kDa dextran by HGF treatment at p41. Data presented as mean ± SEM. Statistical comparisons were made using unpaired *t*-test for comparisons between CTR and EXP groups for 4 kDa conditions (** *p* <0.05). For the 10 kDa conditions, the Mann–Whitney U test was used to compare CTR and EXP groups (** *p* < 0.05). Normality of data distribution was evaluated using the Shapiro–Wilk test Data are shown as mean ± standard error of mean (SEM), sample size of independent experiments *n* ≥ 3.

In order to quantitatively estimate the integrity of the endothelial barrier after HGF treatment, we measured the apparent permeability Transendothelial dextran permeability (TEDP) of the MBEC monolayers by fluorescence-labeled dextrans of increasing molecular sizes (4, 10, 70 KDa). The analysis of paracellular permeability in p5 MBEC (Figure 10A) showed no statistically significant differences between the CTR and EXP-groups. The barrier and permeability characteristics of the CTR group (Figure 10B) exhibited size-selective barrier properties in accordance with a restrictive and functional TJ network. After HGF treatment, both 4 kDa and 10 kDa dextran had significantly greater permeability compared to the CTR group (*p* < 0.01 for both), which demonstrated disrupted paracellular barrier integrity. The permeability to 70 kDa dextran was low and unchanged in all conditions, suggesting that even after HGF application, the monolayer retained a partial barrier function for larger macromolecules at p41.

## 4. Discussion

Despite the considerable number of prior studies that have documented the biological effects of HGF [65,66,67], its precise impact on the endothelial continuum, particularly in the context of endothelial integrity of the blood–brain barrier, remains poorly characterized. This lack of clarity has contributed to a certain degree of conceptual dualism and conflicting interpretations in clinical and experimental approaches. Previous reports have described HGF as a mediator of neuroprotection and angiogenesis [68,69] as well as an endothelial barrier destabilizer, provoking the increased endothelial barrier permeability and endothelial disruption [70]. Such contradictions highlight the complexity of HGF biology and demonstrate the need for more context-specific, multifactorial analyses, which take HGF concentration, time of HGF exposure, tissue environment, and stage of pathological condition into consideration. Based on this, we aimed to investigate the role of HGF as a potential inducer of the MBEC-barrier-like disruption in vitro using primary cell cultures. A specific focus in our experiments was placed on time-dependent differences between early-passage (p5) and late-passage (p41) of primary MBEC cultures. Intermediate time-dependent changes (e.g., passages 15) were not assessed in the current work. Although the sampling across passages was limited, it permitted a clear contrast between an early, stable endothelial state and a late, HGF-induced phenotype.

Our findings indicate that supraphysiological HGF levels can weaken MBEC-barrier-like model properties and promote dysfunction. Several clinically oriented studies reported that HGF may weaken the blood–brain barrier [71,72]. However, in our in vitro experiments, we did not test disease mechanisms or causality. Moreover, all clinical associations cited here are provided solely for context and to generate hypotheses; they do not imply causation. Effects of HGF in supraphysiological doses on the MBEC-barrier-like model may be closely related to the rearrangement of TJ proteins and cytoskeletal remodeling, leading to increased endothelial barrier permeability (leaking endothelial barrier), as observed in our results. We applied a 4 µL/mL HGF exposure as a mechanistic stress-dose to probe the resilience of our MBEC-barrier-like model. It is interesting that high levels of HGF were reported in the literature and are usually associated with a wide spectrum of diseases [73,74,75], pulmonary edema [76], and multiple myeloma [77]. Our in vitro findings are consistent with results in which sustained high HGF levels can weaken properties of the MBEC-barrier-like structure [71,72].

Results shown here prove that microenvironmental cues, such as the aberrantly high levels of HGF, can modulate the morphology and function of cultured primary MBECs in a time-dependent manner. This aspect is closely related to the idea of endothelial plasticity, which was discussed by our team in a previous study [78]. Any phenotypic changes generated by environmental stimuli may underlie both progression and amelioration of endothelial barrier dysfunction. In this research, we did not pursue exhaustive BBB profiling across multiple markers such as claudin-5(CLDN5). Occludin (OCLN) and GLUT1. Our analyses centered on vWf and ZO-1, reflecting endothelial phenotype and TJ integrity.

We observed that ZO-1, a crucial tight junction protein, and vWf, a classical marker of endothelial identity, are differently regulated in response to HGF treatment in a time-and dose-dependent manner. Western blotting detected a notable HGF-dependent reduction in ZO-1 expression, especially in late-passage MBECs (p41), while vWf expression remained stable or slightly upregulated. In concordance with these findings, brightfield microscopy revealed that the HGF treatment provoked a shape shift in MBECs: from a typical elongated, spindle-shaped appearance to a more rounded, less organized phenotype similar to liver endothelial cells. Quantitative circularity analysis confirmed that these morphological changes were significantly more pronounced in late passage MBECs (p41), consistent with cytoskeleton remodeling and loss of a coordinated, tight monolayer structure. These morphological changes were further confirmed and visualized with confocal microscopy, which revealed that ZO-1 was progressively lost at the intercellular borders after HGF exposure, accompanied by disruption of cortical F-actin organization (shown by reduced colocalization with phalloidin staining). All data together may indicate that the junctional complex is decoupled from the actin cytoskeleton, which can be considered a hallmark of barrier destabilization. Beyond the spatial changes of ZO-1, functional assays (TEER and TEDP) demonstrate barrier weakening under HGF. To assess these functional changes coinciding with the altered morphology, we performed a physiological set of experiments with TEER and TEDP assays. Functionally, the structural disorganization was accompanied by a pronounced decrease in TEER and an increase in paracellular permeability with dextran molecules of different sizes. The increased TEDP of the endothelial monolayer was most evident for small (4 kDa) and intermediate (10 kDa) dextran, while larger molecules (70 kDa) remained largely excluded, indicating selective TJ impairment. Absolute TEER values in our model are lower than those reported for optimized human iPSC-BMEC systems (≥1000 Ω·cm^2^) but are consistent with the lower TEER regime typically observed in primary murine BBB cultures [79,80]. Thus, our conclusions are based on within-model CTR–HGF comparison, not on absolute physiological equivalence. Matched passage controls show that passage alone does not influence phenotype changes: at p41 CTR, MBECs retain spindle morphology, membrane-associated ZO-1, and functionally demonstrate the higher TEER. MBECs p41 with supraphysiological concentrations of HGF exhibit spheroid morphology, near-absent ZO-1, markedly reduced TEER, and increased 4–10 kDa dextran permeability.

Taken together, our data provide compelling evidence that in our MBEC-barrier-like model, the supraphysiological levels of HGF promote TJ disassembly, cytoskeletal remodeling, and increase paracellular permeability, particularly in late-passage endothelial monolayers (p41). Based on this, we speculate that chronically elevated concentrations of HGF in the bloodstream may also destabilize an endothelial brain barrier. The enhanced reactivity of endothelial barriers may be driven not only by increased HGF concentrations but also by long exposure time, especially in aging endothelial cells. These speculations are supported by the fact that HGF is a physiologically active endogenous growth factor with a dual nature. On the one hand, it is crucially involved in morphogenesis and organ development [81,82,83], non-pathological liver and body homeostasis [84,85,86], inflammatory regulation [87,88,89], and regeneration [90,91,92]. Under supraphysiological concentrations, on the other hand, it can initiate or amplify a pathophysiological cascade (in a time- and dose-dependent manner) that contributes to severe endothelial barrier dysfunction. Based on the literature review, persistently elevated HGF levels have been associated with various liver diseases, including chronic and acute liver failure, liver cirrhosis of different etiology, and chronic viral hepatitis [93,94,95,96,97], demonstrating at the same time a positive correlation with markers of liver injury [98]. Most patients with liver diseases in their terminal stages tend to develop cerebral edema, hepatic encephalopathy, and other systemic vascular complications, phenomena which may potentially be linked, at least in part, to HGF-mediated endothelial barrier disruption [99]. However, not only liver pathologies may be related to increased HGF levels, but also Alzheimer’s [100,101], and Parkinson’s [102] diseases, as well as stroke [103] and even coronary heart disease [104].

Another interesting aspect of our MBEC-barrier-like model is related to vWf expression. Dushpanova et al. [105] reported weakened blood barrier properties which may be related to vWf-NOX signaling. The authors found that endothelial vWf knockdown dampens NOX-mediated ROS and ET-1 during Ang II stimulation, indicating that endothelial vWf can modulate NOX activity. In our experiments, vWF levels did not change, indicating a vWF-independent mechanism. However, HGF can exert context-dependent, bidirectional effects on endothelial redox status-activating NOX2/H2O2 signaling in some models [105,106], yet suppressing ROS and stabilizing junctions in others [107]. Additionally, the model used by Dushpanova et al. is not directly comparable to ours. We studied MBECs, an MBEC-barrier-like model, whereas Dushpanova’s findings were obtained in porcine aortic endothelial cells. Although both are endothelial cells, they differ markedly in function and phenotype (e.g., tight junction organization, shear-stress phenotype, baseline transcytosis, and inflammatory/oxidative signaling profiles). Thus, any divergence between our results and those of Dushpanova et al. likely reflects vascular bed-specific biology rather than a true contradiction.

The main limitations which we faced in this study were as follows: (1) In vitro model application: this strategy may not fully reflect the complexity of the endothelial barrier in the living organism. (2) Transcriptomic profiling and BBB markers: herein, we did not quantify additional BBB markers CLDN5/OCLN/Glut-1 or perform transcriptomic profiling; thus, we refrain from asserting global BBB marker loss, and future studies will incorporate these assays to delineate the molecular programs underlying the HGF responses. (3) Application of supraphysiological HGF concentration: HGF content in the blood can be different in human disease, which makes an extrapolation of findings to clinical conditions very difficult. (4) We did not measure canonical senescence markers nor include intermediate passages e.g., passage 15. Thus, we cannot exclude any contributions from culture aging. We assume that a continued vWf expression indicates preserved endothelial identity. However, in our future studies, we will quantify senescence markers and validate the effects of HGF on intermediate passages (e.g., *p* ≤ 15). (5) TEER measurements: TEER is sensitive to species, co-culture, media supplements, shear stress, electrode geometry, and temperature. While our static primary mouse model demonstrates lower absolute TEER, we provide convergent evidence of barrier weakening via ZO-1 junctional disassembly and size-selective tracer permeability, in line with recommended practice to pair TEER with paracellular flux. (6) Mechanistic links to neurovascular complications: we did not assess mechanistic links to hepatic encephalopathy, cerebral edema, or other neurovascular disorders; however, prior studies indicate an association between these conditions and elevated HGF levels. (7) Link with oxidative stress: we did not measure oxidative stress, ROS, or NADPH oxidase activity. Although BBB literature implicates ROS in TJ remodeling and HGF–ROS crosstalk [18,19,20], our conclusions are based on structural-functional readouts (ZO-1 mis-localizations; TEER decrease, size-selective permeability) rather than ROS inference. Future work will test NOX2/NOX4 and ROS involvement.

## 5. Conclusions

Our findings revealed significant morphological and physiological changes in MBECs exposed to supraphysiological doses of human HGF. This may suggest a possible cross-organ phenotypic shift (transition) from a brain-specific to a liver-like phenotype. The results were supported by the loss of tight junction integrity and the acquisition of characteristics typical of sinusoidal mouse liver endothelial cells (MLECs) [108,109,110]. Moreover, the MBEC monolayers treated with supraphysiological doses of HGF functionally increased their permeability, which may reflect a transition from a highly regulated MBEC-barrier-like endothelial phenotype toward a “leakier” endothelial profile, typical of hepatic vasculature. This supports the idea that environmental stimuli, such as prolonged HGF exposure, can reprogram brain endothelial cells toward a liver-like phenotype, both structurally and functionally. This experimental in vitro study on the MBEC-barrier-like model provides the first insights for developing possible endothelial barrier destabilization. According to our concept, this may happen through the cytoskeletal decoupling and TJ disassembly. Interestingly, our data may also indicate an amplified vulnerability of endothelial cells to HGF, brought about by prolonged cell passaging, making the endothelial barrier especially susceptible to systemic HGF influence over time.

## Data Availability

The data presented in this study are available on request from the corresponding author.

## Abbreviations

Alpha-SMA	alpha 2 smooth muscle actin
BBB	Blood–Brain Barrier
Cadmec	Dermal microvascular cells
ECs	endothelial cells
EHT	endothelial to hematopoietic transition
EMT	endothelial to mesenchymal transition
FSP1	fibroblast-specific protein-1
GATA-2	binding protein 2
HGF	hepatocyte growth factor
HSCs	hematopoietic stem cells
MBEC(s)	mouse brain endothelial cell(s)
MLEC(s)	mouse liver endothelial cells
RUNX1	runt-related transcription factor-1
TAL1	T-cell acute lymphocytic leukemia protein-1
Tie2	tyrosine kinase with immunoglobulin-like and EGF-like domains
TJs	tight junctions
VE	cadherin-vascular endothelial cadherin
vWf	von Willebrand factor, Anti-factor VIII
